# Adherence to Statin Among Diabetic Patients in Diabetic Centers in Qassim Region, Saudi Arabia

**DOI:** 10.7759/cureus.46742

**Published:** 2023-10-09

**Authors:** Mariam S Alharbi, Shoug Alnasyan, Ghayda Almazroa, Fai N Aldakheel, Ghaida A Albattah, Atheer H AlHujilan

**Affiliations:** 1 Department of Internal Medicine, College of Medicine, Qassim University, Buraydah, SAU; 2 College of Medicine, Qassim University, Buraydah, SAU; 3 Department of Endocrinology, Endocrinology and Diabetes Center, King Fahad Specialist Hospital, Buraydah, SAU

**Keywords:** diabetes mellitus, endocrine, dyslipidemia, diabetes mellitus(dm), medications adherence, statin use

## Abstract

Background

Dyslipidemia affects approximately one-third of Saudi Arabia’s adult population. Dyslipidemia, hypertension, diabetes mellitus (DM), smoking, and a familial predisposition to cardiovascular disease (CVD) are significant risk factors for CVD. It can be prevented effectively through lifestyle changes and lifelong statin therapy; however, poor adherence limits its effectiveness. This study is designed to assess the level of adherence to statin prescription in patients with DM in diabetic centers in the Qassim region and to assess the factors associated with neglecting to take medication.

Methodology

A cross-sectional study was conducted among 226 diabetic patients who were prescribed statins. Medication adherence was assessed using the eight-item Morisky Medication Adherence Scale (MMAS-8). Demographic and clinical data were collected, and multivariate logistic regression analysis was used to identify factors associated with medication adherence.

Results

Of the 226 patients, 29.7% had high adherence, 32.7% had medium adherence, and 37.6% had low adherence to statin medication. Patients diagnosed with diabetes for less than five years had the highest proportion of low adherence (41.2%). No significant associations were found between medication adherence and gender, nationality, or educational level.

Conclusion

The study found that medication adherence to statins in diabetic patients in the Al Qassim region of Saudi Arabia is suboptimal, with a significant proportion of patients having low adherence. Patients diagnosed with diabetes for less than five years had the highest proportion of low adherence, suggesting that patients with a shorter disease duration may require additional support or interventions to improve their medication adherence. Healthcare providers should emphasize the importance of medication adherence and work with patients to develop personalized treatment plans that include medication and lifestyle modifications to optimize lipid control and improve overall health outcomes in diabetic patients.

## Introduction

Cardiovascular disease (CVD) is the leading cause of death in the world [1]. Approximately 3.8 million men and 3.4 million women die from coronary heart disease (CHD) each year, making it the leading cause of premature death worldwide [2]. The risk of CHD increases with hypercholesterolemia [3]. Dyslipidemia, hypertension, diabetes mellitus (DM), smoking, and a familial predisposition to CVD are significant risk factors for CVD. CVD can be prevented effectively through lifestyle changes and lifelong statin therapy; however, poor adherence limits its effectiveness [3]. In primary and secondary prevention, hypolipidemic agents reduce the risk of cardiovascular events. Clinical outcomes for cardiovascular patients are strongly influenced by compliance with lipid-lowering therapy [4]. According to the World Health Organization, non-adherence to long-term medication use is a common problem associated with conditions like hypertension, dyslipidemia, and diabetes, which have severe economic consequences due to wasting time and money and incurring uncurable diseases [5].

Since cardiovascular events and overall mortality are markedly higher in people with established coronary artery disease (i.e., secondary prevention), they must be identified and targeted in risk management strategies [6,7]. Lifelong statin therapy is recommended by evidence-based guidelines for primary prevention in patients with a high-risk profile for CVD and secondary prevention in patients with established CVD [8,9]. An individual's medication adherence is defined in terms of how closely their behavior matches the healthcare provider's recommendations [5]. In a retrospective analysis of 21,239 new statin therapy users, statin-adherent patients were more likely to reach their therapeutic goal of lowering low-density lipoprotein (LDL) cholesterol levels during the first 90 days after starting statin therapy [10]. Understanding the factors that increase the risk of medication non-adherence, given the impact of non-adherence on healthcare outcomes and costs, is essential [11]. In developed countries, including the United States, approximately 50% of patients with chronic diseases do not adhere to their medication regimens [5]. Population-based observational studies from the United States, Canada, and Taiwan have shown suboptimal adherence to statin therapy [12-15]. A study in Kuwait found poor statin adherence to be common, especially among young patients. The CHD risk profile in these patients and statin adherence are inversely correlated [16]. However, a Saudi study in Riyadh involving 1532 diabetic patients revealed that 77% were considered adherent and about 42% achieved the LDL cholesterol goal of 2.6 mmol/L [17].

Dyslipidemia affects approximately one-third of Saudi Arabia's adult population (over 18 years) [18-20]. Information about treatment adherence and persistence, as well as their potential influences, is essential to improving healthcare delivery. Currently, limited information on adherence to statin medication and its related factors among Saudi patients with high CVD risk is available. Therefore, our study aims to evaluate statin adherence among high-risk patients and associated factors in the Al Qassim region of Saudi Arabia.

## Materials and methods

This study aimed to conduct a retrospective, cross-sectional, descriptive study at diabetic centers in the Qassim region to assess medication adherence among diabetic patients who were prescribed statins. The study was conducted from December 2022 to May 2023. The study population consisted of diabetic patients who had been prescribed statins and presented at diabetic centers in the Qassim region. The sample size was calculated based on the expected prevalence of 50% and an absolute error of 5%, resulting in a sample size of 384.

The inclusion criteria for the study were diabetic patients who had a statin prescription, patients who agreed to participate, male and female patients, and diabetic patients above 18 years old who presented in diabetic centers in the Qassim region. The exclusion criteria were diabetic patients who did not have a statin prescription, patients who did not agree to participate, diabetic patients below 18 years old, and diabetic patients who were not presented in diabetic centers in the Qassim region.

Data were collected from diabetic outpatient visitors by taking their consent and distributing an Arabic questionnaire about drug adherence using the Morisky Medication Adherence Scale (MMAS-8), which was a self-reported, medication-adherence questionnaire divided into three levels, with a score of 8 denoting high adherence, 6 to <8 denoting medium adherence, and <6 denoting low adherence. Then, the lipid profile was checked for each participant from medical records in addition to the patient’s demographic data (age, gender, nationality, educational level) and other variables related to diabetes (type, duration of disease, family history, drugs used as treatment, microvascular complications of disease, diabetes diet, exercise). We also asked about factors that could affect adherence, including the presence of hypertension, previous heart disease if present, part of the day the patient took medication, and fear of side effects.

A pilot study was conducted to validate the questionnaire by randomly selecting people from the community, testing the questionnaire, and editing the questions to a more clear form. The other part of the questionnaire was a previously published and validated Arabic MMAS-8. The pilot study helped us to identify any potential problems with the questionnaire and refine it further to ensure that it was easily understandable and could be completed by the respondents without any difficulty. The feedback obtained from the pilot study was used to modify the questionnaire before starting the actual study.

The study used appropriate statistical tests to explore the association between medication adherence and various demographic and clinical factors. Data were analyzed using the Statistical Package for the Social Sciences (SPSS; IBM Corp., Armonk, NY). We measured the p-value and the 95% confidence intervals. The p-value < 0.05 was taken as the fixed point for statistical significance. The study also explored the association between medication adherence and various demographic and clinical factors using appropriate statistical tests. We applied for ethical approval from the Qassim Research Ethics Committee (QREC) before conducting the study. Survey data were kept entirely confidential, and only researchers were able to access the survey's data. Participants were informed that participation in the study was voluntary and that they could withdraw from the study at any time without any consequences. We also ensured that the study adhered to the principles of the Declaration of Helsinki and other relevant ethical guidelines.

## Results

Table 1 displays the demographic factors of the patients included in the study. The majority of the patients were over 50 years old (81.4%), and 18.6% were under 50 years old. The gender distribution was slightly skewed toward females, with 61.1% of the patients being female and 38.9% being male. Almost all of the patients were Saudi nationals (97.8%), with only 2.2% being non-Saudi. The educational level of the patients varied, with the highest proportion being uneducated (35.4%), followed by primary school (21.7%), university (19.0%), high school (12.8%), and middle school (11.1%).

**Table 1:** Demographic factors of the patients

		Count	%
Age	<50 years old	42	18.6%
	>50 years old	184	81.4%
Gender	Male	88	38.9%
	Female	138	61.1%
Nationality	Saudi	221	97.8%
	Non-Saudi	5	2.2%
Educational level	Uneducated	80	35.4%
	Primary school	49	21.7%
	Middle school	25	11.1%
	High school	29	12.8%
	University	43	19.0%

Table 2 displays the characteristics of DM and other baseline health-related conditions of the patients included in the study. The majority of patients had type 2 diabetes (83.2%), with 16.8% having type 1 diabetes. Most patients had been diagnosed with diabetes for more than 10 years (61.1%), while 17.3% had been diagnosed for less than five years and 21.7% had been diagnosed for five to 10 years. A family history of diabetes was present in 70.8% of patients. Almost all patients (99.6%) were taking diabetes medications, with the majority taking a combination of oral glycemic drugs and injections (34.1%). A little over half of the patients (57.5%) were committed to a diabetic diet, and a similar proportion of patients (56.5%) reported exercising at least three to four times a week. A quarter of the patients (25.2%) reported suffering from heart disease, while 63.3% reported having high blood pressure. The majority of patients (60.2%) reported taking their medications both during the day and at night, while 24.8% reported taking their medications at night and 15.0% during the day. Over four-fifths of patients (82.3%) reported not avoiding taking statins medication for high cholesterol due to fear of side effects, while 17.7% reported avoiding it.

**Table 2:** Characteristics of diabetes mellitus and other baseline health-related conditions

		Count	%
Type of diabetes	Type 1	38	16.8%
	Type 2	188	83.2%
Duration of diabetes	<5 years	39	17.3%
	5-10 years	49	21.7%
	>10 years	138	61.1%
Family history of diabetes mellitus	No	66	29.2%
	Yes	160	70.8%
Does the patient use diabetes medications?	No	1	0.4%
	Yes	225	99.6%
Anti-diabetic medication	None	1	0.4%
	Oral glycemic drugs	88	38.9%
	Injection	60	26.5%
	Oral glycemic drugs and injection	77	34.1%
Is the patient committed to a diabetic diet?	No	96	42.5%
	Yes	130	57.5%
Does the patient exercise?	No	84	37.2%
	Less than one monthly	10	4.4%
	1-2 times monthly	29	12.8%
	3-4 times weekly‎	60	26.5%
	Daily	43	19.0%
Does the patient suffer from heart disease?	No	169	74.8%
	Yes	57	25.2%
Does the patient suffer from high blood pressure?	No	83	36.7%
	Yes	143	63.3%
What time does the patient take the medications?	Day	34	15.0%
	Night	56	24.8%
	Both	136	60.2%
Does the patient not take statins (medicine for high cholesterol) because of fear of side effects?	No	186	82.3%
	Yes	40	17.7%

**Figure 1:**
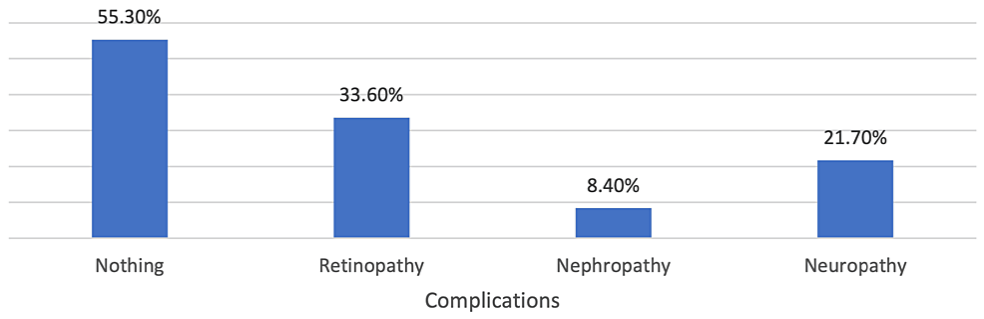
Complications related to diabetes mellitus

Figure 1 displays the percentage of cases with complications related to DM among the patients included in the study. The majority of patients (55.3%) did not report any complications related to DM. Among those who did, the most common complication reported was retinopathy, which was present in 33.6% of cases. Neuropathy was reported in 21.7% of cases, while nephropathy was present in only 8.4% of cases.

**Figure 2:**
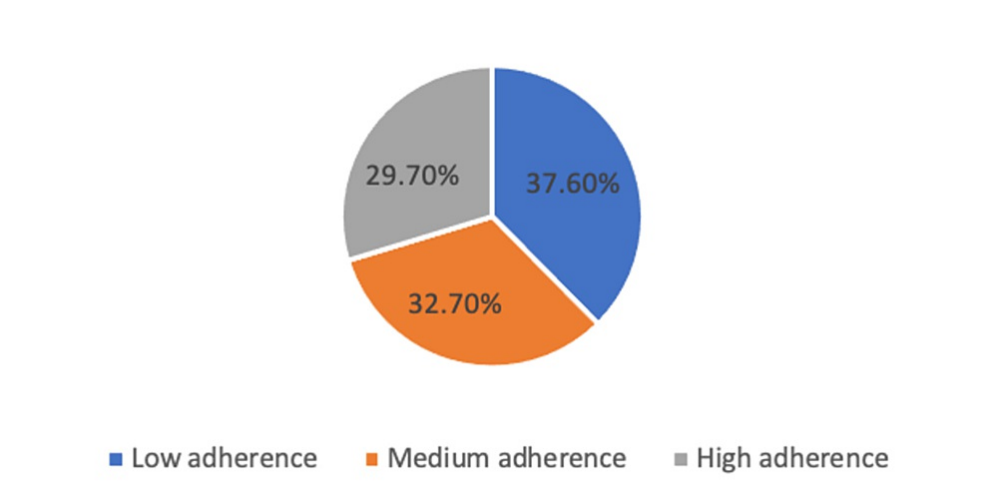
The distribution of the patients according to adherence to statin

Figure 2 displays the distribution of patients according to their adherence to statin medication. Of the total patients (n = 226), 37.6% had low adherence, 32.7% had medium adherence, and 29.7% had high adherence to their statin medication regimen.

Table 3 presents the association between adherence to statin medication and the demographic factors of the patients included in the study. The analysis shows that age was significantly associated with adherence to statin medication (p = 0.009), with patients under 50 years old having a higher proportion of low adherence (54.8%) compared to patients over 50 years old (33.7%). No significant associations were found between gender, nationality, or educational level and adherence to statin medication.

**Table 3:** The association between adherence to statins and the demographic factors of patients * Significant at p-value lower than 0.05.

		Adherence						
		Low adherence		Medium adherence		High adherence		P-value
		Count	%	Count	%	Count	%	
Age	<50 years old	23	54.8%	14	33.3%	5	11.9%	0.009*
	>50 years old	62	33.7%	60	32.6%	62	33.7%	
Gender	Male	35	39.8%	30	34.1%	23	26.1%	0.651
	Female	50	36.2%	44	31.9%	44	31.9%	
Nationality	Saudi	83	37.6%	73	33.0%	65	29.4%	0.800
	Non-Saudi	2	40.0%	1	20.0%	2	40.0%	
Educational level	Uneducated	29	36.3%	25	31.3%	26	32.5%	0.861
	Primary school	17	34.7%	14	28.6%	18	36.7%	
	Middle school	10	40.0%	10	40.0%	5	20.0%	
	High school	12	41.4%	11	37.9%	6	20.7%	
	University	17	39.5%	14	32.6%	12	27.9%	

Table 4 presents the relationship between adherence to statin medication and DM and other health-related conditions among the patients included in the study. The analysis shows that the duration of diabetes was significantly associated with adherence to statin medication (p = 0.029), with patients diagnosed with diabetes for less than five years having the highest proportion of low adherence (48.7%). No significant associations were found between type of diabetes, family history of diabetes, anti-diabetic medication, commitment to a diabetic diet, exercise, heart disease, high blood pressure, time of medication intake, or fear of side effects and adherence to statin medication.

**Table 4:** The relation between adherence to statins and diabetes mellitus and other health-related conditions * Significant at p-value lower than 0.05.

		Adherence						
		Low adherence		Medium adherence		High adherence		P-value
		Count	%	Count	%	Count	%	
Type of diabetes	Type 1	10	26.3%	17	44.7%	11	28.9%	0.168
	Type 2	75	39.9%	57	30.3%	56	29.8%	
Duration of diabetes	<5 years	19	48.7%	12	30.8%	8	20.5%	0.029*
	5-10 years	25	51.0%	10	20.4%	14	28.6%	
	>10 years	41	29.7%	52	37.7%	45	32.6%	
Family history of diabetes mellitus	No	21	31.8%	25	37.9%	20	30.3%	0.450
	Yes	64	40.0%	49	30.6%	47	29.4%	
Anti-diabetic medication	None	1	100.0%	0	0.0%	0	0.0%	0.081
	Oral glycemic drugs	41	46.6%	20	22.7%	27	30.7%	
	Injection	16	26.7%	27	45.0%	17	28.3%	
	Oral glycemic drugs and injection	27	35.1%	27	35.1%	23	29.9%	
Is the patient committed to a diabetic diet?	No	41	42.7%	28	29.2%	27	28.1%	0.380
	Yes	44	33.8%	46	35.4%	40	30.8%	
Does the patient exercise?	No	28	33.3%	27	32.1%	29	34.5%	0.602
	Less than one monthly	5	50.0%	4	40.0%	1	10.0%	
	1-2 times monthly	10	34.5%	9	31.0%	10	34.5%	
	3-4 times weekly‎	24	40.0%	17	28.3%	19	31.7%	
	Daily	18	41.9%	17	39.5%	8	18.6%	
Does the patient suffer from heart disease?	No	66	39.1%	53	31.4%	50	29.6%	0.683
	Yes	19	33.3%	21	36.8%	17	29.8%	
Does the patient suffer from high blood pressure?	No	34	41.0%	26	31.3%	23	27.7%	0.727
	Yes	51	35.7%	48	33.6%	44	30.8%	
What time does the patient take the medications?	Day	18	52.9%	8	23.5%	8	23.5%	0.301
	Night	22	39.3%	19	33.9%	15	26.8%	
	Both	45	33.1%	47	34.6%	44	32.4%	
Does the patient not take statins (medicine for high cholesterol) because of fear of side effects?	No	65	34.9%	62	33.3%	59	31.7%	0.164
	Yes	20	50.0%	12	30.0%	8	20.0%	

Table 5 reports the effect of adherence to statin medication on the lipid profile of the patients included in the study. The table displays the mean and standard deviation of cholesterol, LDL, HDL, and triglycerides (TG) for patients with low adherence, medium adherence, and high adherence to statin medication, as well as for all patients combined. The analysis shows that patients with high adherence to statin medication had lower mean levels of cholesterol (3.80 mmol/L) and LDL (2.40 mmol/L) compared to patients with low adherence (4.57 mmol/L and 2.84 mmol/L, respectively) and medium adherence (4.25 mmol/L and 2.67 mmol/L, respectively). The differences in mean levels of cholesterol and LDL between high adherence and low adherence were statistically significant (p = 0.001 and p = 0.025, respectively). No significant differences were found in mean levels of HDL or triglycerides between adherence groups.

**Table 5:** Report of the effect of adherence to statins on lipid profile of patients * Significant at p-value lower than 0.05. LDL: low-density lipoprotein; HDL: high-density lipoprotein; TG: triglycerides.

Adherence		Cholesterol	LDL	HDL	TG
Low adherence	Mean	4.57	2.84	1.25	1.49
	Std. deviation	1.42	1.06	0.58	0.69
Medium adherence	Mean	4.25	2.67	1.1301	1.46
	Std. deviation	1.16	1.02	0.26	0.65
High adherence	Mean	3.80	2.40	1.11	1.46
	Std. deviation	0.93	0.81	0.25	0.64
P-value		0.001*	0.025*	0.062	0.927
Total	Mean	4.24	2.65	1.17	1.47
	Std. deviation	1.24	0.99	0.41	0.66

## Discussion

The purpose of this research was to analyze the factors that contribute to non-adherence to statin therapy among high-risk patients in the Al Qassim area of Saudi Arabia. According to the study's findings, about two-thirds of diabetic individuals who administered statins had low or moderate drug adherence. Of the total number of patients in the study (n = 226), 37.6% had low adherence to their statin prescription regimen, 32.7% had medium adherence, and 29.6% had good adherence. These results are concerning because better health outcomes and the avoidance of diabetes and high cholesterol-related problems depend on patients taking their medications as prescribed. This study's findings on medication adherence in individuals with chronic conditions are in line with those of other researchers. Patients with chronic conditions, such as diabetes and high cholesterol, frequently struggle with prescription non-adherence. Depending on the study group and the technique of assessing adherence, reports of suboptimal medication adherence in diabetic patients range from 30% to 80% [21,22]. Rates of drug adherence in patients with high cholesterol have also been shown in studies to be unsatisfactory, ranging from 30% to 50% [23,24]. Medication adherence studies in other chronic conditions have yielded outcomes similar to those seen in this one. Medication adherence in patients with hypertension, for instance, was found to have a mean of 57%, with rates ranging from 18% to 94%, according to a systematic study [25]. The mean percentage of drug adherence in patients with heart failure was 50%, with rates ranging from 10% to 98% in another systematic analysis [26].

Higher levels of good cholesterol and LDL were linked to improved lipid profiles in diabetes patients who took statins consistently. Patients who took their statins as prescribed had improvements in their cholesterol and LDL levels compared to those who took them less or more often. There were statistically significant variations in the mean levels of cholesterol and LDL between those with strong adherence and those with low adherence. These results stress the significance of regular medication use in helping diabetic people improve their health. Consistent with other research, we found that higher rates of statin drug adherence were related to improved lipid profiles in diabetes individuals [17,27]. Statin therapy dramatically lowers LDL cholesterol levels in people with diabetes, resulting in a decreased risk of cardiovascular disease and mortality, as shown by a comprehensive review and meta-analysis of randomized controlled trials [6]. Good control of LDL cholesterol levels has been linked to better health outcomes in patients with diabetes, according to a number of studies [28,29].

There is a lot of evidence showing that reducing LDL and total cholesterol levels is crucial for diabetes individuals [30,31]. The leading cause of morbidity and mortality in diabetic individuals is cardiovascular disease, and high levels of LDL and total cholesterol are key risk factors for this condition [32]. Heart attack, stroke, and peripheral vascular disease are just some of the issues that can come from atherosclerosis, which can be brought on by elevated LDL and total cholesterol levels [33]. Reducing the risk of these issues and improving the overall quality of life in diabetes individuals can be achieved by proper control of LDL and total cholesterol levels through medication adherence and lifestyle adjustments such as diet and exercise. Uncontrolled levels of LDL and total cholesterol in diabetes people can increase the risk of heart disease and also contribute to other issues [34,35]. For instance, diabetic retinopathy, a condition that can cause blindness, has been linked to elevated levels of LDL cholesterol [36]. Diabetic nephropathy, a condition that can lead to kidney failure, has also been linked to elevated total cholesterol levels [37].

Patients diagnosed with diabetes for less than five years had the highest proportion of low adherence to statin medication, according to the study. This finding suggests that patients diagnosed with diabetes for shorter durations may benefit from additional support or interventions to improve their adherence to statin medication. Consistent with other research, we found that individuals with a diabetes diagnosis of less than five years had the highest proportion of low adherence to statin therapy. Several studies have shown that people with a recent diabetes diagnosis are less likely to take their medications as prescribed [38,39]. Reasons for this may include unfamiliarity with the condition and its treatment, aversion to potential adverse effects, and trouble adapting to a new medication schedule, among others. Adherence to statin medicine was not shown to be influenced by gender, country of origin, or level of education. Some research has found that people of different sexes, ethnicities, and educational backgrounds are less likely to take their medications as prescribed [40,41]. Demographic characteristics may not be as important as age, socioeconomic position, and co-morbidities in determining drug adherence [25].

Although this study sheds light on statin adherence among diabetic patients in the Al Qassim region of Saudi Arabia, it is not without flaws that should be taken into account. The results cannot be extrapolated to the broader population because of the limited sample size and single-center methodology. The inability to evaluate trends in medication compliance over time or establish causality is another limitation of a cross-sectional study design. Lack of information on reasons for non-adherence and patient characteristics may also restrict the interpretability of the data, as may the dependence on self-reported drug adherence, which may be biased. The study's findings on medication adherence in this demographic, however, are important and can help guide the creation of focused interventions to boost medication compliance and, in turn, improve health outcomes for diabetic patients. Larger sample sizes, longitudinal designs, and gathering more thorough information on patient characteristics and causes for non-adherence are all ways that future research can improve on the current study's limitations.

## Conclusions

In conclusion, the findings of this study contribute significantly to our understanding of medication adherence among diabetic patients in the Al Qassim region of Saudi Arabia who were administered statins. These results underline the need for focused interventions to increase diabetes medication adherence, particularly among individuals with a recent diabetes diagnosis. Better health outcomes for diabetes patients are a primary focus of the study, which also highlights the significance of physician adherence to recommendations. Medication adherence among diabetic patients could be improved by future studies that investigate the causes of non-adherence and design specific interventions to address these issues.
